# Supplementary material for: The discharge process for a child with complex healthcare needs dependent on respiratory technology: A scoping review protocol
Source: PLoS One. 2026 May 27;21(5):e0349670. doi: 10.1371/journal.pone.0349670 (PMC13215509; doi:10.1371/journal.pone.0349670)
Supplement: S2 File — (DOCX) [file pone.0349670.s002.docx]

***CINAHL Search Strategy***

1. TI ( Discharg* OR “discharge plan*” OR “discharge process*” OR “patient discharge” OR transfer* OR “discharge management” OR transition OR “care transition*” OR “hospital discharge” OR “discharge instruction*” OR “discharge pathway*” OR “discharge education” OR “discharge planning from hospital to home” OR “alternate level of care” OR “delay* transfer of care” ) OR AB ( Discharg* OR “discharge plan*” OR “discharge process*” OR “patient discharge” OR transfer* OR “discharge management” OR transition OR “care transition*” OR “hospital discharge” OR “discharge instruction*” OR “discharge pathway*” OR “discharge education” OR “discharge planning from hospital to home” OR “alternate level of care” OR “delay* transfer of care”)
2. (MH "Transfer, Discharge") OR (MH "Patient Discharge") OR (MH "Discharge Planning") OR (MH "Patient Discharge Education")
3. S1 OR S2
4. TI ( Child* OR adolescen* OR youth OR “young person” OR teenager OR kids OR p#ediatric* OR “young adult” OR infant ) OR AB ( Child* OR adolescen* OR youth OR “young person” OR teenager OR kids OR p#ediatric* OR “young adult” OR infant )
5. (MH "Infant, Newborn")
6. S4 or S5
7. TI ( "complex healthcare needs” OR chronic critical illness" OR “complex needs” OR “special need*” OR “complex care need*” or “complex care” OR “complex care issues” OR “complexity of healthcare needs” OR “complex and challenging health care needs” OR “complex physical conditions” OR “chronic condition*” OR “long term conditions” or “chronic disease” OR “chronically ill” OR “complicated chronic condition* OR ‘crippled child* OR “cyborg” OR “congenital and critical illness” OR “complex health care needs” OR “complex health needs” OR “complex respiratory health care needs” OR “complex conditions” OR “complex conditions” OR “life limiting conditions” OR “complex medical issues” OR “chronic conditions” OR “chronic illness” OR “complex or homecare services” OR “complex medical conditions” OR “complex medical care” OR “complex medical needs” OR “complex medical conditions” OR “complex chronic condition*” OR “complexity of care” OR “care complexity” OR “health care vulnerabilities” OR “exceptional healthcare needs” OR “life-limit* illness*” OR “life-threatening condition” OR “life-shortening conditions” OR “life-altering chronic illness” OR “medical complexity” OR “medically complex” OR “medically fragile” OR “medically complex chronic condition*” OR “medical fragility” OR “neurodisability” OR “clinical fragility” OR “p#diatric palliative care” OR “end of life care” OR “sick child*” OR “ special healthcare needs” OR “special needs” ) OR AB ( "complex healthcare needs” OR chronic critical illness" OR “complex needs” OR “special need*” OR “complex care need*” or “complex care” OR “complex care issues” OR “complexity of healthcare needs” OR “complex and challenging health care needs” OR “complex physical conditions” OR “chronic condition*” OR “long term conditions” or “chronic disease” OR “chronically ill” OR “complicated chronic condition* OR ‘crippled child* OR “cyborg” OR “congenital and critical illness” OR “complex health care needs” OR “complex health needs” OR “complex respiratory health care needs” OR “complex conditions” OR “complex conditions” OR “life limiting conditions” OR “complex medical issues” OR “chronic conditions” OR “chronic illness” OR “complex or homecare services” OR “complex medical conditions” OR “complex medical care” OR “complex medical needs” OR “complex medical conditions” OR “complex chronic condition*” OR “complexity of care” OR “care complexity” OR “health care vulnerabilities” OR “exceptional healthcare needs” OR “life-limit* illness*” OR “life-threatening condition” OR “life-shortening conditions” OR “life-altering chronic illness” OR “medical complexity” OR “medically complex” OR “medically fragile” OR “medically complex chronic condition*” OR “medical fragility” OR “neurodisability” OR “clinical fragility” OR “p#diatric palliative care” OR “end of life care” OR “sick child*” OR “ special healthcare needs” OR “special needs” )
8. TI ( “Respiratory technology” OR “Technology dependen*” OR “Technological dependen*” OR “medical technology” OR “Life threatening dependen* on technology” OR “Trach*” OR “long term ventilation” OR “chronic ventilation” ) OR AB ( “Respiratory technology” OR “Technology dependen*” OR “Technological dependen*” OR “medical technology” OR “Life threatening dependen* on technology” OR “Trach*” OR “long term ventilation” OR “chronic ventilation” ) =
9. (MH “technology, medical”)
10. S8 OR S9
11. S3 AND S6 AND S7 AND S10
12. Limits: peer review, English and 2015 - 2025
