# Supplementary material for: The discharge process for a child with complex healthcare needs dependent on respiratory technology: A scoping review protocol
Source: PLoS One. 2026 May 27;21(5):e0349670. doi: 10.1371/journal.pone.0349670 (PMC13215509; doi:10.1371/journal.pone.0349670)
Supplement: Attachment — Submitted filename: S3 Response to reviewers.docx [file pone.0349670.s005.docx]

**Reply to reviewer’s comments**

We would like to thank the editor and the reviewers for their comments. All comments were taken into consideration and changed as per the table below and the line reference number is for the version with no tracking in it for clarity.

| **Revisions requested** | **Response** |
| --- | --- |
| The abstract reads more like a general summary than a structured scientific abstract. It lacks subheadings (Objective, Introduction, Methods, etc.), which are preferred for protocols. | The layout for the abstract was changed in the last submission as per the editor’s request (Background, objectives, methods, expected results, conclusion) (line 29 – 67) |
| Key methodological parameters (e.g., inclusion/exclusion rationale, expected number of reviewers, data charting tools, timeline) are mentioned but not sufficiently detailed. | This has been amended as per your recommendation.  Five reviewers will be involved throughout all stages of screening, of screening and all stages will be completed by two independent reviewers to enhance accuracy and reduce bias (line188 - 190).  Data will be extracted from each article using a standardised data extraction tool in Covidence for evidence synthesis (line 193 – 194).  The timeline for the review is specified as per the recommendation  ‘The schedule of screening record screening to be completed by November 2025, data extraction by February 2026 and full completion of scoping review by April 2026’. (line 140 - 142). |
| The inclusion of the word “protocol” in the short title is appropriate but could be accompanied by “scoping review protocol following JBI and PRISMA-ScR” for indexing precision. | This has been changed to reflect this recommendation  ‘a scoping review protocol following JBI and PRISMA-ScR guidelines’  (line 4) |
| Clarify the timeframe (“since June 2015”) and justify it (e.g., relevance to advances in complex care or technology-dependent paediatrics). | This has been changed to reflect the recommendation. ‘The timeframe chosen reflects advances in nursing and medical care, pharmacological and medical technology, and greater life expectancy’  (line 169 - 170). |
| The introduction is overly descriptive and literature-heavy, sometimes blurring the line between background and results of prior reviews. Some references (e.g., U.S. and Irish studies on discharge delays) are cited without explicit interpretation or critical comparison. The problem statement could be sharper in identifying the gap in existing evidence synthesis (e.g., absence of scoping reviews mapping this process). | This has changed to include more specific information from the US and Irish studies (Line 88 - 99). |
| There are minor inconsistencies in referencing format (e.g., punctuation, spacing around years and semicolons). | References have been updated to be uniform. |
| What you should do is to condense background data (lines 83–150) to focus on the rationale for conducting a scoping rather than a systematic review. | The background has been cut significantly to be more comprehensive |
| Explicitly state the knowledge gap, for instance: “Although discharge processes for children with medical complexity have been described in narrative or single-centre studies, no comprehensive synthesis of factors influencing discharge for respiratory technology-dependent children exists.” | This statement has been included before the aim of the study. (line 111 - 113) |
| Link the introduction directly to how this protocol addresses practice or policy gaps (e.g., informing discharge pathway design, as mentioned on line 155). | This has been amended to directly link the gap in practice and policy.  The aim of this scoping review is to explore the current discharge process for children with complex healthcare needs who are dependent on respiratory technology. This will be necessary to inform subsequent planned research to create a discharge pathway for children dependent on respiratory technologies to reduce hospital stay and to complement the national framework for children with complex healthcare needs’  (line 113 - 118) |
| Objectives could be mapped to the PCC (Population–Concept–Context) structure for alignment with JBI guidance. | The objectives have been updated to reflect this recommendation. (line 124 - 132). |
| The last objective (“What constitutes an effective discharge…”) should specify the operational definition of “effective”, e.g., timely, safe, family-centred. Present the objectives using PCC format. | Objective four has been reworded for clarity using the PCC format. (line 131 - 132). |
| Define “respiratory technology” (invasive/non-invasive ventilation, tracheostomy, long-term oxygen) early in the objectives. | Respiratory technology has been defined as per the recommendation as per the US Congress Office of Technology Assessment  (Line 74 – 77) |
| As far as the search strategy is concerned: “Search terms were systematically identified” (line 190), but lack any example of search strings or keywords. They should at least summarise the key concepts (children, complex healthcare needs, respiratory technology, discharge, transition). | This has been amended as per the recommendation  ‘Search terms were systematically identified through a rigorous process, which highlighted a total of 43 different terms used internationally for children with complex healthcare needs’  (line 156 - 159) |
| My advice is to summarise the search strategy within the text and include Boolean logic example (“complex healthcare needs” AND “respiratory technology” AND “discharge”). | This has been amended as per your recommendation  ‘Additional searches related to discharge and respiratory technology will also be completed, combined with ‘AND’ and is available in supplement 2 for full details’  (line 160 - 161). |
| Supplement S2 is cited but not briefly described in the text. | This has been amended as per the recommendation ‘the search strategy for the CINAHL database including the four main terms in either title or abstract, MeSH subject headings, Boolean operators and study limitations applied are fully detailed and available in supplemental 2’  (line 162 - 165). |
| Language restriction to English may exclude relevant studies from non-English European countries. | A substantial body of research has been undertaken (pending publication) which highlighted most publications have been in published in the English language, despite English not being the main spoken language of the country, but unfortunately owing to PLOS One guidelines, it cannot be included. |
| Exclusion of grey literature (line 245) is a notable limitation given that discharge process data often arise from policy documents, hospital reports, or theses. | The authors are aware of this limitation, and it is noted in line 227 - 228. |
| There is no stakeholder consultation described, which JBI recommends as an optional but valuable step.  Add a short statement acknowledging the potential contribution of consultation with stakeholders (families, clinicians, discharge coordinators). | The authors acknowledge that stakeholder consultation is a valuable step to all healthcare related literature reviews and the authors plan to have both PPI and expert contribution during the subsequent parts of this doctoral work and PPPI contribution in the next steps is discussed (line 234 – 238) |
| In the data charting form content is listed (line 216–221) but not illustrated. | This has been further clarified ‘Data will be extracted from each included article, using a standardised data extraction tool in Covidence for evidence synthesis’  (Line 193 - 194) |
| In the analysis “Narrative synthesis” (line 234) is appropriate but should specify which analytical approach will be applied (e.g., thematic synthesis, mapping by micro/meso/macro levels). | This is further clarified as per the recommendation ‘Study characteristics will be categorized and presented in micro (patient-level) meso (institutional) and macro (policy / system) factors’  (Line 210 – 211) |
| Ethics, although correctly stated as not required, could be briefly mentioned in the Methods for completeness. | This has been added as per the recommendation into the methods section (line 54 – 55). |
| Clarify analytic framework—e.g., “Data will be thematically categorized according to micro (patient-level), meso (institutional), and macro (policy/system) factors.” | This sentence has been added into the data analysis and presentation section  ‘Data will be thematically categorised according to micro) patient-level), meso (institutional) and macro (policy / system) factors’  (line 210 - 211 |
| In the Results section, there is no plan for handling missing or incomplete data described (e.g., contacting authors). | This has been amended as per the recommendation  ‘If full articles aren’t available through the databases, the corresponding author will be contacted for the full text’  (line 175 – 176) |
| It also lacks mention of software for managing extracted data (e.g., Excel, NVivo). | This has been amended as per the recommendation  ‘Data will be extracted from each included article, using a standardised data extraction tool in Covidence for evidence synthesis’ (line 193 - 194) |
| The protocol omits a plan for charting the number of studies by design, country, and year, which is expected in scoping reviews  Include statement: “Data will be charted descriptively by study type, country, setting, and design.” | This has been amended as per the recommendation.  ‘Data will be charted descriptively by title, authors, year of publication, country of publication and or where research was conducted, aims / purpose, population and sample size, methodology, setting, discharge pathway, timeframes of discharge process, process and outcomes measures, contextual factors that hinder and facilitate discharges, and key findings in relation to scoping review questions’  (line 194 – 199) |
| Specify that results will be summarised via tables and thematic charts (consistent with JBI format). | This has been amended as per the recommendation  ‘Information related to the research questions will be summarized using tables and thematic charts consistent with JBI guidelines, completed with a narrative synthesis’  (line 210 - 211 |
| Add a brief plan for quality assurance, even if critical appraisal is not required for scoping reviews. | This has been expanded as per the recommendation  ‘there will be five reviewers, and all stages of screening will be completed by two independent reviewers which will enhance accuracy and reduce bias’ (line 188 - 190) |
| The discussion (lines 238–249) is brief and reads more like a reiteration of the purpose than an analytical reflection. | This has been clarified and elaborated as per your recommendation  ‘This scoping review protocol outlines a comprehensive synthesis of peer-reviewed literature examining the initial hospital-to-home discharge process for children with complex healthcare needs who are dependent on respiratory technology, with the aim of identifying critical gaps, challenges, and opportunities for improvement. The factors identified through the review will be used for a proposed discharge pathway for children with complex healthcare needs dependent on respiratory technology  (line 220 - 225). |
| It could elaborate on how findings will inform practice (e.g., hospital discharge protocols) and policy (e.g., national complex care strategies). | This had been amended to add in the following line  ‘findings from the scoping review will inform the next steps of research to consider the development of a discharge pathway within the context of the national policy of integrated care and care closer to home  (line 232 - 238). |
| It does not mention a dissemination plan beyond publication (“will be published in a peer-reviewed journal”). | This has been amended to clarify dissemination plans  ‘Findings from this scoping review will be disseminated through peer-reviewed journals, national and international conferences, and both formal and informal teaching platforms to reach key stakeholders, policymakers, and clinicians (line 232 - 234). |
| I recommend that you expand the discussion to clarify anticipated contributions to practice, research, and policy. | This has been amended as per the recommendation  ‘Findings from the scoping review will inform the next step of research which will focus on the development of a discharge pathway. This will be undertaken in partnership with patient and public involvement stakeholders situated within the context of national policy priorities on integrated and delivering care closer to home’  (line 232 – 238) |
| Also, suggest inclusion of how the resulting map will support families or inform future interventions. You can add a note on planned dissemination (e.g., conference presentation, stakeholder summary). | This has been amended as per the recommendation  ‘Findings from the scoping review will inform the next step of research which will focus on the development of a discharge pathway. This will be undertaken in partnership with patient and public involvement stakeholders situated within the context of national policy priorities on integrated and delivering care closer to home’  (line 232 - 238) |
| There is inconsistent formatting in the references (e.g., some missing periods or irregular capitalisation; Pediatric Otolaryngology vs Journal of Pediatrics). | This has been rectified throughout the protocol as per the recommendation |
| Some DOIs are missing, though PLOS requires them. | This has been rectified throughout the reference list as per the recommendation, however, some documents referenced are national and International documents or ebooks that do not have DOIs and therefore Vancouver style referencing was applied as per PLOS One guidelines. |
| References 12–15 include mixed styles (numbering before sentence punctuation). | This has been rectified as per the recommendation. |
| Please revise references to conform strictly to PLOS ONE’s numeric citation style with DOIs for all journal articles. | This has been rectified as per the recommendation |
| Consider reducing redundancy (some overlapping citations for delayed discharge). | References have been reduced as per the recommendation |
| There are several grammatical issues and inconsistent tense (e.g., “The inclusion criteria is presented…” → should be “are presented”). | This has been rectified throughout the protocol |
| There is an overuse of long sentences, which reduces clarity. | Thank you for this suggestion and this has been rectified throughout the protocol. |
| There is inconsistent use of “children with complex healthcare needs” vs. “children with medical complexity.” | This has been rectified throughout the document to ensure consistency of terminology. |
| The “Data Availability” statement (page 5) repeats standard text without specifying a repository plan. | The statement on page 5 is a standard sentence as per PLOS one during the submission process. |
| Include a short forward-looking statement: “All extracted data and synthesised tables will be made available in an open-access repository (e.g., Zenodo, Open Science Framework) upon publication.” | This has been added in as per the recommendation  ‘all extracted data and synthesis tables will be made available in CORA, the authors university of employment, which supports the deposit of the final manuscript version of publication for open access publication’  (line 215 – 217) |
