# Supplementary material for: The discharge process for a child with complex healthcare needs dependent on respiratory technology: A scoping review protocol
Source: PLoS One. 2026 May 27;21(5):e0349670. doi: 10.1371/journal.pone.0349670 (PMC13215509; doi:10.1371/journal.pone.0349670)
Supplement: Attachment — Submitted filename: S4 Response to reviewers.docx [file pone.0349670.s006.docx]

**Reply to reviewer’s comments**

We would like to thank the editor and the reviewers for their comments. All comments were taken into consideration and changed as per the table below and the line reference number is for the version with no tracking in it for clarity.

| **Revisions requested** | **Response** |
| --- | --- |
| 1)Although Reviewer 2 recommended adding ‘A scoping review protocol following JBI and PRISMA‑ScR Guidelines’ as the short title, I recommend keeping the shorter version: ‘A scoping review protocol. | Thank you for your suggestion, this has been amended to  ‘The discharge process for a child with complex healthcare needs dependent on respiratory technology: A scoping review protocol’  Line 2 - 3 |
| 2)The statement ‘Data extracted will include information on key findings related to the aim and objectives of the scoping review’ can be moved to the following sentence, as the main focus of the preceding statement is on the review and screening procedures, followed by data extraction.” | Thank you for your suggestion, this has been amended to  Two reviewers will review each stage with further discussion as required for exclusion and inclusion. Data extracted will include information on key findings related to the aim and objectives of the scoping review and will be summarized and presented in tables and charts with a narrative summary.  Line 52 - 55 |
| 3) It is not common practice to report the ethical statement in the abstract. According to the PLOS ONE guidelines, it should instead be included under the ‘Ethics Statement’ subheading. | Thank you for your suggestion, this has been amended under a separate subheading.  ‘Ethical approval has not been sought for the present study because it is a review of published academic literature’  Line 271 - 273 |
| 4) Rephrase the expected results statements for better clarity and avoid redundancy consider the following ideas : what the review will produce, how the results will be organized( i.e., Evidence will be organised across micro‑level (child and family), meso‑level (healthcare team and institutional processes), and macro‑level (policy and system) influences., and what type of knowledge the review will generate. | Thank you for your suggestion, this has been amended to  ‘International guidelines on care closer to home and caring for children with tracheostomies state the need for discharge pathways. This review will synthesis existing literature regarding the discharge processes of children with complex healthcare needs dependent on respiratory technology. Evidence will be organised across micro-level (child and family), meso-level (healthcare team and institutional processes), and macro-level (policy and system) influences. This scoping review will identify the multifaceted barriers and facilitator that impact the transition from hospital to home for children dependent on respiratory technology’  Line 58 - 67 |
| 5) Redundancy in terminology has been noted. For example, the term ‘healthcare needs’ is repeated within the same sentence in lines 71–73, and the word ‘technologies’ appears redundantly in lines 76–78. Please consider removing such repetition to improve readability and flow. | Thank you for your suggestion, this has been amended to remove redundant words.  Line 78-80 has been removed.  Respiratory technologies has been removed from line 82  The paragraph has been edited to  ‘A child with complex healthcare needs is considered to have one or more complex chronic condition, substantial healthcare needs, functional limitations, and significant healthcare usage [1]. A child dependent on medical technology requires a device or devices to provide a vital body function, and can include tracheostomies, invasive or non-invasive ventilation and oxygen [4]. There continues to be international inconsistences in the terminology used to describe children with complex healthcare needs, resulting in reported prevalence rates ranging from 0.2% - 11.4%’  Line 76 - 88 |
| 6) Consider rewriting the statement in lines 78–80 as follows to improve clarity: “There continue to be international inconsistencies in the terminology used to describe children with complex healthcare needs, resulting in reported prevalence rates ranging from 0.2% to 11.4%.” | Thank you for your suggestion, this has been amended to  ‘There continue to be international inconsistencies in the terminology used to describe children with complex healthcare needs, resulting in reported prevalence rates ranging from 0.2% to 11.4%.’  Line 85 - 88 |
| 7) For line 86, consider rephrasing “A delay in discharge is when” to: “A delay in discharge occurs when a patient…” | Thank you for your suggestion, this has been amended to  ‘A delay in discharge occurs when a patient is deemed medically fit to be discharged to but to operational or logistical issues, they remain in hospital’  Line 89 - 90 |
| 8) . For improved readability, consider rewriting the statements in lines 90–95 as follows: “Across four US hospital centres, discharge delays were observed in 68.5% (n=37) of children, with an average length of stay of 53.9 days (range 4–204 days). Most of these cases (n=31) involved children with tracheostomy tubes.” | Thank you for your suggestion, this has been amended to  ‘Across four US hospital centres, discharge delays were observed in 68.5% (n=37) of children, with an average length of stay of 53.9 days (range 4–204 days). Most of these cases (n=31) involved children with tracheostomy tubes’  Line 94 - 96 |
| 9) Line 94-101: Consider removing the notation (n=8 or 13 etc)’ or replacing it with a written expression to improve readability and flow. | Thank you for your suggestion, this has been amended to  ‘Evidence from the UK and Ireland demonstrates significant discharge delays for children with complex healthcare needs. In a UK tertiary centre, delays of over 3 months were noted in 50% (n=8) of admissions, while an Irish study reported average hospitalisations of 8.7 months (range 3-22 months) despite medial stability’  Line 105 - 109 |
| 10) In lines 103–104, the phrase ‘can impact on early childhood development and’ may be removed to avoid redundancy and improve clarity. | Thank you for your suggestion, this has been amended to  ‘Prolonged stays in hospital for children with long-term health problems are at a greater risk of development delays due to lack of age-appropriate stimulation and by lack of social interaction and gaining age-appropriate skills’  Line 117 - 120 |
| 11) Lines 115–116: Consider rephrasing ‘The aim of this scoping review is to explore’ to ‘This scoping review therefore aims to…’ to improve flow and alignment with the preceding sentence. 1213. 14.. | Thank you for your suggestion, this has been amended to  ‘the scoping review therefore aims to explore the current discharge process for children with complex healthcare needs who are dependent on respiratory technology’  Line 129 - 132 |
| 12) Lines 117–120: This section would benefit from revision to more clearly emphasise the specific advantages for children who are dependent on respiratory technologies. | Thank you for your suggestion, this has been amended to  ‘This review will provide the necessary foundation for future research aimed at developing a discharge pathway for children dependent on respiratory technology. This pathway seeks to minimize hospital stays and align with the national framework for children with complex healthcare needs’  Line 135 – 138. |
| 13) Lines 138 and 140: Delete the phrase ‘hereafter known as’; using the abbreviation (JBI) alone is sufficient. | Thank you for your suggestion, this has been amended to  ‘Joanna Briggs Institute (JBI)’  Line 155 - 156 |
| 14) The statement in line 142-144” rephrase “The screening of records is scheduled for completion by November 2025, followed by data extraction by February 2026, with full completion of the scoping review expected by April 2026 | Thank you for your suggestion, this has been amended to  ‘The screening of records is scheduled for completion by November 2025, followed by data extraction by February 2026, with full completion of the scoping review expected by April 2026’  Line 160 - 162 |
| 15) Statement line 145-146: According to the PLOS ONE guidelines, it should be included under the ‘Ethics Statement’ subheading. | Thank you for your suggestion, this has been amended under a separate subheading.  ‘Ethical approval has not been sought for the present study because it is a review of published academic literature’  Line 271 - 273 |
| 16. Table 1 lacks clarity, and the following points should be addressed to improve accuracy and alignment with the PCC framework: a. Remove Redundancy: There is no need to repeat “Young people >18 years of age” in the exclusion criteria, as the age range is already clearly defined in the inclusion criteria. b. Rephrase and Clarify Statements Under Inclusion Criteria – Concept: 1. Studies examining the discharge process for children dependent on respiratory technology. 2. Studies reporting process related or clinical outcomes at the micro (patient), meso (healthcare setting), or macro (policy/societal) levels c. Rephrase and Clarify Statements Under Exclusion Criteria – Concept: 1. Studies not addressing discharge processes or related clinical outcomes 2. Studies focusing on subsequent admissions or discharges after the initial discharge home d. Clarify Context Criteria: Under Inclusion Criteria: 1. Discharge pathways from hospital to home, including intermediate care settings 2. Consider clarifying whether discharges from community or home care settings to home are also included or excluded, as this is currently ambiguous. 3. Peer reviewed primary research from any geographical setting e. Remove or Relocate Unnecessary Narrative Content i. The following statement in Table 1 lacks clarity and appears out of place: “Setting from hospital to home may include several intermediate settings. In a retrospective review in the US from 2016–2019, 22% of children were discharged to other facilities [19].” This information is descriptive rather than a criterion and should be removed from the table. f. The inclusion and exclusion criteria related to study type—such as opinion pieces, letters to the editor, non–peer‑reviewed publications, and language restrictions—can be added to Table 1 under a new column titled ‘Additional Eligibility Criteria. | Thank you for your suggestion, this has been amended to  a. removed from exclusion criteria  b. rephrased as per your recommendation  c. rephrased as per your recommendation  d. rephrased as per your recommendation  e. This has been removed as per your recommendation  f. This has been amended as per your recommendation and a 4^th^ column added titled ‘additional eligibility criteria’ |
| 17) The paragraph “Types of sources” (lines 152–156) can be removed if the authors decide to add an “Additional Eligibility Criteria” column to Table 1 | Thank you for your suggestion, this has been amended with an additional column has been added to the inclusion exclusion table.  Line 171 |
| 18) Lines 178–179 should be rephrased as follows: “The corresponding author will be contacted for any missing full texts or data.” Consider the repetition in line 209-210. It is important to clearly state how missing information will be handled. | Thank you for your suggestion, this has been amended to  ‘If full articles are not available through databases, the corresponding author will be contacted for any missing full texts or data’.  Line 198 - 200 |
| 19) The statements in line 176-177 is duplicated with statement 197-198 related to using Covidence tool with 2 different references please reconcile and remove duplicate | This duplication has been removed and all references checked |
| 20) The statement in lines 185–186 is duplicated in lines 189 regarding the involvement of a third reviewer. Please remove the duplication to ensure consistency and avoid repetition. The same issue appears with the statements in lines 181 and 192–194. I recommend reviewing the entire paragraph to improve readability and eliminate repetitive content. | Thank you for your suggestion, this has been amended to  ‘Five reviewers will be involved throughout all stages of screening, and all stages will be completed by two independent reviewers to enhance accuracy and reduce bias. Titles / abstracts will be screened independently by two reviewers. During this phase, each reviewer will independently assess the titles and abstracts of the articles identified in the search. Reviewers will classify each record as ‘yes’ (meets inclusion criteria), ‘maybe’ (uncertain or requires further discussion) or ‘no’ (does not met inclusion criteria). Any discrepancies will be reviewed and resolved through a discussion with a third reviewer at each stage of screening and data extraction. A PRISMA ScR flow chart will be included to illustrate the study selection process, including reasons for exclusion and results from each stage of screening’  Line 202 - 211 |
| **21)** Line 220: Please ensure that the abbreviation “CORA” is defined at first use. If it has already been defined earlier in the manuscript, avoid repeating the full term and use only the abbreviation. | Thank you for your suggestion, this has been amended to  ‘All extracted data and synthesis tables will be made available in Cork Open Research Repository (CORA)’  Line 244 |
| **22)** Please add the language restriction as a study limitation. Limiting the review to English‑language studies should be explicitly acknowledged under the “Limitations” section | Thank you for your suggestion, this has been amended to  ‘A further limitation of this study is the inclusion only of English-language literature which may introduce geographical and linguistic bias’.  Line 259 - 261 |
