# Supplementary material for: The discharge process for a child with complex healthcare needs dependent on respiratory technology: A scoping review protocol
Source: PLoS One. 2026 May 27;21(5):e0349670. doi: 10.1371/journal.pone.0349670 (PMC13215509; doi:10.1371/journal.pone.0349670)
Supplement: Attachment — Submitted filename: Reviewer Comments to the Author.docx [file pone.0349670.s003.docx]

Reviewer Comments to the Author

Thank you for the opportunity to review your protocol titled *“The discharge process for a child with complex healthcare needs dependent on respiratory technology.”* This is a well-conceived and well-written paper that addresses an important and often overlooked aspect of pediatric complex care. The topic is timely, and the proposed scoping review has clear potential to inform better discharge planning and continuity of care for children who depend on respiratory technologies.

Overall, the manuscript follows an appropriate structure and aligns with JBI and PRISMA-ScR methodology. The introduction provides a comprehensive overview of the issue, and the rationale for the review is well established. The objectives are generally clear and relevant to the stated aim.

A few points for revision before acceptance:

1. Objective 5 Missing:
   In the “Objectives” section, numbering stops at point 4, and Objective 5 is left blank. Please review and clarify whether an additional objective was intended or if this was a formatting oversight.
2. Data Availability Statement:
   Please include a short note about where the data from this review will be made available once completed (for example, through a public repository or upon reasonable request).
3. Clarify the Review Question:
   The main review question might be rephrased slightly to emphasize exploration, for instance: “What is known about the discharge processes for children with complex healthcare needs who are dependent on respiratory technology?” This wording better fits the scoping review approach.
4. Framework Alignment:
   Your eligibility criteria table currently uses the Population–Exposure–Outcome (PEO) format. JBI guidance recommends the Population–Concept–Context (PCC) framework for scoping reviews. Adjusting this would align the protocol with standard practice.
5. Ethical Transparency:
   Please include a statement confirming that ethical approval was not required, as the review will use publicly available data and will not involve human participants.
6. Terminology and Consistency:
   Define and consistently use terms such as “children with complex healthcare needs” versus “children with medical complexity” to avoid ambiguity.
7. Minor Editing Suggestions:
   - Define all abbreviations (e.g., JBI, PRISMA-ScR) at first use.
   - Break longer paragraphs into shorter ones for readability.

Consider briefly outlining how you plan to disseminate findings to key audiences (e.g., clinicians, policymakers, families). A visual summary of the discharge process at micro, meso, and macro levels might also add clarity when publishing the final review.

No ethical or publication concerns are identified. The proposed methodology is rigorous and feasible.

Overall recommendation: *Accept with minor revisions.*
This is a thoughtful, well-organized protocol that will make a valuable contribution once the minor issues above are addressed, particularly the clarification of the missing objective and inclusion of a data availability statement.
