# Supplementary material for: The discharge process for a child with complex healthcare needs dependent on respiratory technology: A scoping review protocol
Source: PLoS One. 2026 May 27;21(5):e0349670. doi: 10.1371/journal.pone.0349670 (PMC13215509; doi:10.1371/journal.pone.0349670)
Supplement: Attachment — Submitted filename: Reply to reviewer comments 18.12.25.docx [file pone.0349670.s004.docx]

**Reply to reviewer’s comments**

We would like to thank the editor and the reviewers for their comments. All comments were taken into consideration and changed as per the table below.

| **Revisions requested** | **Response** |
| --- | --- |
| Reformat the abstract into a structured version (Background, Objectives, Methods, Expected Results, Conclusion). | Move introduction piece and renamed it background (line 30 – 38)  Results and Conclusion added line 53 – 62.  Removed inclusion criteria from abstract and restructured as per recommendation. |
| Replace the PEO framework with the PCC (Population–Concept–Context) format, in accordance with JBI guidance. | Line 177 (table one)  Study eligibility framework revised with reference included to population, concept and contexts |
| Correct the numbering of objectives  clearly define “effective discharge” and “respiratory technology.” | This has been rectified. There is no objective 5, it was a typographical error.  Respiratory technology defined in line 69 – 72.  What constitutes an effective discharge (line 160 – 161) is an objective of the scoping review as despite the term ‘effective discharge’ being used it does not appear to have a definition. |
| Include examples of search terms and Boolean logic, justify the 2015 start date, and specify how data will be organized and analyzed (software, thematic framework). | The search strategy has been updated to include examples of search terms and to make the search strategy clearer (Line 186 - 195). |
| Provide a concrete data availability plan (e.g., Zenodo, OSF). | Data accessed is publically available research, therefore no data availability plan is needed.  All data extraction tables will be published as part of the supplementary material or availbale on request. |
| Add a brief ethics statement confirming that no human or identifiable data are involved. | Line 172 – 173  This statement has been added to the methods section confirm no ethical approval was required, using publicly available data with no human participants |
| Expand the discussion to explain how results will inform practice, pathway design, and policy, and briefly mention dissemination plans. | Line 251 – 254 added in for further clarity. However, as the scoping review has not been completed it is not possible to identify what pathway design will be undertaken. |

**Editor’s Revisions**

| **Revisions requested** | **Response** |
| --- | --- |
| The introduction, although comprehensive, could be more focused on the knowledge gap justifying this review, avoiding excessive repetition of descriptive data. | Introduction revised and reduced. |
| Consider registering the protocol in PROSPERO or the Open Science Framework (OSF) to enhance transparency. | Thank you for this suggestion, registration with OSF will be considered |
| Clarify how quality assurance and inter-reviewer agreement will be monitored during screening and data extraction. | Line 206 – 207.  More information added to ensure further clarity around inter-reviewer agreement |
| The manuscript would benefit from shorter paragraphs and clearer transitions to improve readability. | Paragraphs shortened to ease of reading |
| Ensure terminology consistency across all sections—use “children with complex healthcare needs” uniformly. | Line 66 - 68  Sentence restructured to  ‘In several countries children with complex healthcare needs are termed children with medical complexity or children with special healthcare needs and within that term are children who are dependent on medical technology’. For the remainder of the submission, the term children with complex healthcare needs |
| The statement of aims and policy relevance could be strengthened by explicitly linking expected outputs to clinical and system-level decision-making | Line 58 – 59.  Identifies what this scoping review contributes to with stakeholder and public patient involvement |

| **General Comments** | **Response** |
| --- | --- |
| Remove all citations from abstract | All citations removed |
| Minor Editing Suggestions:  Define all abbreviations (e.g., JBI, PRISMA-ScR) at first use. | Line 166 – 167  JBI abbreviation written in full and initials used thereafter.  Line169 - 171  PRISMA ScR abbreviation written in full and initials used thereafter |
| Review the references and language for consistency, grammar, and DOIs. | All references updated as per Vancouver referencing style. |
